# Supplementary material for: Characterization and Antibacterial Activity of Silver Nanoparticles Synthesized from Oxya chinensis sinuosa (Grasshopper) Extract
Source: Microorganisms. 2024 Oct 18;12(10):2089. doi: 10.3390/microorganisms12102089 (PMC11509906; doi:10.3390/microorganisms12102089)
Supplement: Supplementary file 1 [file microorganisms-12-02089-s001.zip › microorganisms-3231672-supplementary.pdf]

Supplementary material

Characterization and Antibacterial Activity of Silver Nanoparticles Synthesized from *Oxya chinensis sinuosa* (Grasshopper) Extract

Se-Min Kim [1,2], Tai-Yong Kim [1], Yun-Sang Choi [3], Gyeongsik Ok [1] and Min-Cheol Lim [1,4,*]

[1] Research Group of Food Safety and Distribution, Korea Food Research Institute, Wanju-gun 55365, Republic of Korea
[2] Department of Food Science and Biotechnology, Chung-Ang University, Anseong-si 17546, Republic of Korea
[3] Research Group of Food Processing, Korea Food Research Institute, Wanju-gun 55365, Republic of Korea
[4] Department of Food Biotechnology, Korea University of Science and Technology, Daejeon-si 34113, Republic of Korea
* Correspondence: mclim@kfri.re.kr; Tel.: +82-62-219-9310

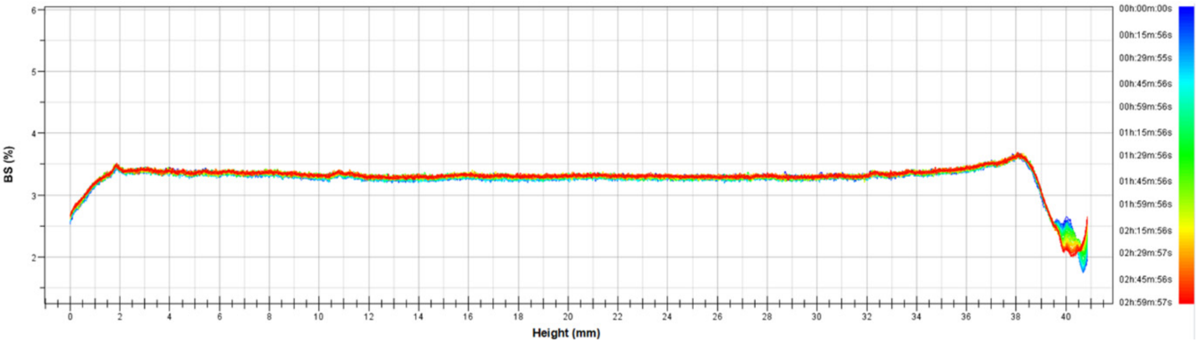

Figure S1. Turbiscan of the AgNPs synthesized using the O_extract. Backscattering profiles (%) of the O_AgNPs
